# But, after all, why is it important to assess the auditory processing?

**DOI:** 10.5935/1808-8694.20130097

**Published:** 2015-10-08

**Authors:** Berenice Dias Ramos

**Affiliations:** Preceptor of the Medical Residency Program in Pediatric Otorhinolaryngology and Phoniatrics of the Otorhinolaryngology and Head and Neck Department, Pontifical Catholic University of Rio Grande do Sul - PUCRS. MSc in Otorhinolaryngology, Medical School of the Federal University of São Paulo.

When we hear a song, when we attend a class or when we talk to someone, countless processes are triggered in order to enjoy the melody, understand what is being explained by the speaker or listen to a conversation and developing an answer. Over the full 24-hour period we are exposed to multiple auditory informations most of them happen simultaneously and our auditory system is responsible for identifying the messages we are interested in, reducing or canceling the interferences which only serves to prevent the comprehension. The performance of these tasks count on central nervous system activity and it is called auditory processing (AP), which refers to the efficiency and effectiveness by which the central nervous system is able to use auditory information. In other words, AP is a specific set of skills a person depends on to understand what he/she is hearing.

These abilities include sound localization, speech recognition in noise, auditory performance with degraded or distorted acoustic speech signals, auditory performance in competing acoustic signals (including dichotic listening), temporal aspects of audition such as identification of brief and rapidly successive acoustic changes in frequency, intensity or duration.

These skills are extremely important in a classroom, for example, where the student must focus attention on what is been said by the teacher and ignore any stimulus that may negatively interfere with listening: conversations between colleagues, chair dragging sounds, steps in the hallway, the fan noise, horns in the street or yelling in the schoolyard. The child with proper functioning of the central auditory nervous system will easily understand the teacher; while those with AP disorders may have difficulties to understand what is being said - which can negatively impact the learning process.

In normal speech, auditory information reaches the nervous system in a very quick succession. Each information processing must happen in few milliseconds in order that we can understand the meaning. A child with temporal AP disorder may have developmental language disorders and learning disabilities. This happens because the discrimination of syllables such as /ba/, /da/, /ga/, /pa/, /ta/, /ka/ should occur in the first 40 milliseconds, which is a period of time too short for those who have difficulties to detect brief acoustic stimuli.

These difficulties can occur at any age and it is assumed that long standing hearing loss is one of the main causes. Much has been published about AP changes due to otitis media, mainly when it occurs during the first two years of life, but we cannot forget that the elderly, as they lose their hearing slowly as they age, may also have difficulties to understand speech, even while using an appropriate hearing aid, if they were fitted long after the hearing loss onset.

Over the past 20 years there has been a huge progress in neurosciences, which has also been reflected in a better understanding of the AP. Knowledge of the location of the regions involved in the processing of speech, language and learning became more accurate thanks to positron emission tomography and functional magnetic resonance imaging. These new technologies, used primarily by researchers, have also identified areas that decode auditory information, the areas that make the phonological, lexical and semantic analysis, as well as the areas that produce the answers. Electrophysiological tests such as ABR, EEG, P300 and magnetoencephalography can measure the time of transmission and processing of such information in the range of milliseconds.

A battery of behavioral tests with high sensitivity and high specificity helps identify the skills affected and program specific management strategies geared to the problem of each patient.

Conventional audiologic evaluation does not reflect the daily life of a classroom, an office, a business meeting, a conference or a party. This may have been the reason for the difficulty we still have to date to help patients who report difficulties in understanding speech and who have normal, or very close to normal, audiometric values. Whenever a patient has complaints which do not match his hearing thresholds, we should consider expanding our investigation using the available diagnostic methods.

